# A Sensor for Spirometric Feedback in Ventilation Maneuvers during Cardiopulmonary Resuscitation Training

**DOI:** 10.3390/s19235095

**Published:** 2019-11-21

**Authors:** Rodolfo Rocha Vieira Leocádio, Alan Kardek Rêgo Segundo, Cibelle Ferreira Louzada

**Affiliations:** 1 Department of Control and Automation Engineering (DECAT), Escola de Minas, Universidade Federal de Ouro Preto (UFOP), Morro do Cruzeiro, 35400-000 Ouro Preto, MG, Brazil; alankardek@ufop.edu.br; 2 Department of Pediatric and Adult Clinic (DECPA), Escola de Medicina, Universidade Federal de Ouro Preto (UFOP), Morro do Cruzeiro, 35400-000 Ouro Preto, MG, Brazil; cibelle.louzada@hotmail.com

**Keywords:** cardiopulmonary resuscitation, sensor, medical mannequins, spirometric techniques

## Abstract

This work proposes adapting an existing sensor and embedding it on mannequins used in cardiopulmonary resuscitation (CPR) training to accurately measure the amount of air supplied to the lungs during ventilation. Mathematical modeling, calibration, and validation of the sensor along with metrology, statistical inference, and spirometry techniques were used as a base for aquiring scientific knowledge of the system. The system directly measures the variable of interest (air volume) and refers to spirometric techniques in the elaboration of its model. This improves the realism of the dummies during the CPR training, because it estimates, in real-time, not only the volume of air entering in the lungs but also the Forced Vital Capacity (FVC), Forced Expiratory Volume (FEVt) and Medium Forced Expiratory Flow (FEF_20–75%_). The validation of the sensor achieved results that address the requirements for this application, that is, the error below 3.4% of full scale. During the spirometric tests, the system presented the measurement results of (305 ± 22, 450 ± 23, 603 ± 24, 751 ± 26, 922 ± 27, 1021 ± 30, 1182 ± 33, 1326 ± 36, 1476 ± 37, 1618 ± 45 and 1786 ± 56) × 10^−6^ m^3^ for reference values of (300, 450, 600, 750, 900, 1050, 1200, 1350, 1500, 1650 and 1800) × 10^−6^ m^3^, respectively. Therefore, considering the spirometry and pressure boundary conditions of the manikin lungs, the system achieves the objective of simulating valid spirometric data for debriefings, that is, there is an agreement between the measurement results when compared to the signal generated by a commercial spirometer (Koko brand). The main advantages that this work presents in relation to the sensors commonly used for this purpose are: (i) the reduced cost, which makes it possible, for the first time, to use a respiratory volume sensor in medical simulators or training dummies; (ii) the direct measurement of air entering the lung using a noninvasive method, which makes it possible to use spirometry parameters to characterize simulated human respiration during the CPR training; and (iii) the measurement of spirometric parameters (FVC, FEVt, and FEF_20–75%_), in real-time, during the CPR training, to achieve optimal ventilation performance. Therefore, the system developed in this work addresses the minimum requirements for the practice of ventilation in the CPR maneuvers and has great potential in several future applications.

## 1. Introduction

Noninvasive methods used to characterize human and animal respiration require advanced techniques and are costly, such as: computed tomography and densitometry [1,2,3,4,5,6], electrical impedance tomography [7,8], magnetic resonance imaging [9,10,11], contrast radiology [12], image ultrasound [13,14], ultrasonic sensors [15,16,17], pulse oscillometry [18,19], electrostatic methods [20], closed circuits with inert gases [21], impedance pneumography and plethysmography [22,23] and electronic noses [24].

One of the procedures used in plethysmography is spirometry, which uses physical concepts to study the air going in and out of the lungs, characterizing human breathing [25,26]. The technique is used to evaluate pulmonary function [1,18], chronic obstructive pulmonary disease [1,27], cystic fibrosis [2], smokers [4], air pollution [28,29,30], hyperinflation [31,32], exposure of particulates such as nanotubes and nanofibers [33] and airway resistance [34], among others.

To perform spirometry, a spirometer is used, which can be: (i) of volume (sealed in water, piston, and bellows [35]); (ii) of flow (differential pressure or pneumotacometers [36], thermistors, Pitot and turbinometers [35]); or (iii) portable [37] of volume or flow [25]. The respiratory volume sensors used in these types of equipment have a high cost and some of them perform indirect measurements, discarding their application in medical simulators or dummies, as in the case of Hamiltonian sensors coupled to differential pressure sensors [38], flow mass sensors [39] and airflow meter [40] rotary or vibratory beam and shell flow meters [41].

Cardiopulmonary resuscitation (CPR) is a recurring practice in medical urgencies and emergencies. CPR is characterized by a set of maneuvers performed in an attempt to reanimate the victim of cardiac and/or respiratory arrest, to restore the heart and lung to normal functions while maintaining the oxygenation of the brain. These procedures provide continuous improvement in the quality of healthcare professional skills by using automated dummies for teaching.

The pioneers in automating CPR dummies [42] used the Resusci Anne^®^ manikin. The authors developed compression and ventilation sensors using digital logic and normally open contacts. The monitoring of the system was done using a display panel or indicator lamps, which showed indications of “insufficient”, “acceptable”, and “above acceptable”. Currently, identical models are still widely used [43].

In the present study, we propose measuring the volume of air supplied to the lungs in rescue ventilation during CPR training using a rotor-type flow sensor with propellers. Also, we develop a theoretical model to make it equivalent to spirometric models. This brings more realism to the dummies and introduces advantages to possible debriefings after various simulations [44].

This work is an extension of [44], (doi:10.3390/ecsa-5-05724), where only the idea was put forward to assess the feasibility of the application. Additionally, in the present article, we perform the validation of the adapted water flow sensor to measure airflow considering concepts of fluid mechanics. Also, we apply spirometric concepts to the results, defining a theoretical model for the curves obtained.

## 2. Mathematical Modeling of Propeller Type Flow Sensors

The analysis of the behavior of any material contained in a finite region of space, control volume, solves many problems involving fluid mechanics [45]. The Reynolds transport theorem ensures that the time rate of change of mass within a system is equal to the sum of the time rate of mass within the control volume (*CV*) and the net flux of mass through the control surface (*CS*), that is,

(1) $$\frac{{\mathrm{DM}}_{\mathrm{sys}}}{\mathrm{Dt}}=\frac{\partial }{\partial t}\int_{\mathrm{CV}}\rho \mathrm{dV}+\int_{\mathrm{CS}}\rho v\cdot \hat{n}\mathrm{dS},$$

where $M_{sys}$ is the mass of the system (kg), ρ is the specific mass of the fluid (kg/m^3^), V is the control volume (m^3^), and v is the velocity vector perpendicular to the differential area dS (m/s).

Using the principle of mass conservation for a system, the material derivative of the mass of the system is

(2) $$M_{sys}=\int_{sys}\rho dV,$$

therefore,

(3) $$\frac{{DM}_{sys}}{Dt}\;=\;0.$$

At permanent regime, the properties at any point in the system remain constant over time, so

(4) $$\frac{\partial }{\partial t}\int_{CV}\rho dV\;=\;0.$$

Applying (3) and (4) to (1) and adding up all the differential contributions that exist on the control surface, we obtain the net flux of mass in the control volume, that is,

(5) $$\int_{\mathrm{CS}}\rho v\cdot \hat{n}\mathrm{dS}=\sum {\dot{m}}_{O}-\sum {\dot{m}}_{I}=0.$$

Considering that the input of the sensor in question has the same characteristics of the output, that is, $S_{I}=S_{O}$, and applying Equation (3) to its control volume, we conclude that:

(6) $${\dot{m}}_{I}={\dot{m}}_{O}.$$

A widely used expression for mass flow assessment $\dot{m}$ (kg/s), in a section of the control surface with area S (m^2^), is

(7) $$\dot{m}=\rho Q=\rho \mathrm{Sv},$$

where Q is the volume flow (m^3^/s), and v is the velocity vector perpendicular to area S (m/s).

We can adequately analyze many mechanical fluid problems considering a fixed and undeformable control volume. In addition, considering a uniform distribution of the specific mass of the fluid in each flow section (of the compressible flows) allows specific mass variations to occur only from one section to another.

Substituting (7) into (6), we obtain

(8) $$v_{I}=\frac{\rho_{O}}{\rho_{I}}v_{O}.$$

An ideal gas can be characterized by having a large number of molecules, considered as spherical beads with a mass greater than zero and negligible individual volume when compared to the volume containing them [46]. Thus, the evident macroscopic properties of an ideal gas are consequences mainly of the independent movement of the molecules as a whole.

In various conditions the gases deviate from ideality, being characterized as a real gas, which is constituted by particles endowed with chaotic movement, and subjected to the forces of attraction of long-distance and forces of repulsion at a short distance. It is important to know the specific mass range in which an ideal gas equation describes the behavior of one real gas with adequate accuracy. It is important also to know how much the behavior of a real gas can deviate from the ideal gas at a given pressure and temperature. This information originates from the compressibility factor Z. When it is an ideal gas (Z=1), the distance from Z of the unit is a measure of the behavior deviation of the actual gas from that predicted by the ideal gas equation [47]

(9) PV=nRT,

where P is the absolute pressure of the gas (Pa), V is the volume occupied by the gas (m^3^), n is the number of moles of the gas (mols), R is the ideal gas constant (8.31 J/(mol·K)) and T is the temperature (K).

If the temperature ranges from 250 K up to 400 K, and at a pressure of 101,325 Pa, atmospheric air (compressible fluid) approaches to an ideal gas with acceptable accuracy for the system of this work [48]. If the pressure and temperature differences are small, generally less than 10%, air can be considered incompressible. Therefore, we can write Equation (8) as:

(10) $$v_{I}=v_{O}.$$

## 3. Materials and Methods

### 3.1. YF-S201 Flow Sensor

The flow sensor YF-S201 (Sea brand) has been widely used to measure water flow in pipes. It consists of a valve, containing inside it a propeller rotor and a Hall effect sensor, which is commonly used by supply companies to monitor water consumption [43,44,45,46,47]. The rotor has a toroidal magnet that produces an alternating magnetic field as the rotor rotates [49]. The magnetic field interacts with the Hall effect sensor, which in turn produces digital pulses that correspond to the rotor speed. The rotor speed corresponds to the average speed water flowing through the valve [50,51].

Unlike other applications involving YF-S201 sensor, this work is the first one which uses it for measuring air volume and performs spirometric feedback in ventilation maneuvers during CPR using medical simulators or training manikins, in real-time.

**Figure 1:**
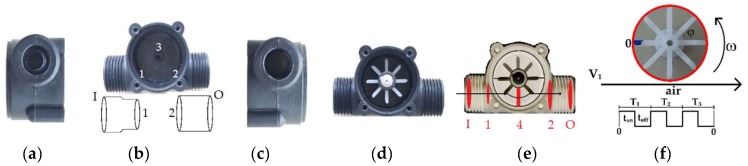
YF-S201 flow sensor: (**a**) Input profile; (**b**) Control volume; (**c**) Output profile; (**d**) Propeller compartment; (**e**) Airflow profile; (**f**) Detail of the propeller.

Figure 1 shows the different views of the sensor. According to Figure 1a–c, there is throttling in the diameter of the sensor inlet channel (the area I relative to 1). Also, there is no difference in the diameter of the output channel (the area 2 relative to O) of the YF-S201 sensor. Therefore, considering the model presented in Section (2) and starting from Equation (10), we can write

(11) $$v_{I}=v_{O}=v_{2}.$$

According to the details of the control volume, shown in Figure 1e, the mass flow in section I is a function of mass flows in Sections 1, 4 and 2. Considering Equation (5) and the flow in the permanent regime, the volume flow of I, 1, 4, and 2 are constants, that is, $v_{1}=v_{4}=v_{2}$ and the rate of temporal variation of the mass contained in the control volume results in:

(12) $${\dot{m}}_{I}={\dot{m}}_{1}+{\dot{m}}_{4}+{\dot{m}}_{2}.$$

Substituting Equation (7) into Equation (12), we obtain

(13) $$v_{I}=\left(S_{1}+S_{4}+S_{2}\right)\frac{v_{1}}{S_{I}}.$$

In Figure 1e we can see that

(14) $$S_{1}=\frac{{\pi d}_{1}^{2}}{4},$$

(15) $$S_{2}=\frac{{\pi d}_{2}^{2}}{4}$$

and

(16) $$S_{4}=\mathrm{bh};$$

where b is the base (m), and h is the height (m) of area 4 oriented entering the plane of the paper in Figure 1e. Thus, the input flow is

(17) $$Q_{\mathrm{IO}}=Q_{I}=S_{I}v_{I}.$$

Substituting Equations (13)–(16) into Equation (17) we found

(18) $$Q_{\mathrm{IO}}=k_{1}v_{1},$$

where $k_{1}$ is a constant which depends on the areas, that is, the geometry of the sensor.

Figure 1d shows the propeller of the YF-S201 sensor, which rotates according to the flow of air passing through it. Figure 1f shows the detail of the propeller, which has a Hall sensor for providing digital pulses proportional to its angular velocity. An ATmega328 microcontroller measures the digital pulses using external interrupt, along with a real-time scheduling and multitasking software [52].

Besides of the Hall effect sensors being widely used in fluids flow measurements [53,54,55], they are also used as magnetic sensors [56,57,58] in numerous applications such in water pump flow measurement [41], infiltrometers [49], energy monitoring [59], electromagnetic flowmeters used in industrial and physiological techniques [60], hydrometers [61], induction-frequency converters [62], among others. 

To make the sensor suitable for measuring airflow, we use the relation of the linear velocity $v_{1}$ (m/s) with the angular velocity ω (rad/s) [47], this is

(19) $$v_{1}=\frac{d_{h}f}{2},$$

where $d_{h}$ (m) is the diameter of the helix and f (H_Z_) is the rotation frequency of the helix.

Applying Equation (19) in Equation (18), we have

(20) $$Q_{IO}=kf$$

where

(21) $$k=d_{h}\left[\frac{\pi }{8}\left(d_{1}^{2}+d_{2}^{2}\right)+\frac{bh}{2}\right]$$

is a constant equal to (261 ± 3) × 10^−8^ m^3^, calculated according to the sensor dimensions. The calculation of the geometric constant k, according to the mathematical modeling presented in this work (Equation (21)), is the first step to adjust the sensor output signal to the unit of measurement: flow (m^3^/s).

From (20), the volumetric flow is

(22) $$V=Q_{IO}\cdot ∆t,$$

where *V* is the volume of air (m^3^) flowing inside the lung (reservoir) of the dummy during the time interval *∆t* (s).

### 3.2. Calibration

After calculating the geometric constant k, according to Equation (21), a calibration procedure was performed to verify the degree of agreement between the measurements made by the YF-S201 sensor and the reference values of (300 ± 2, 450 ± 3, 600 ± 3, 750 ± 4, 900 ± 5, 1050 ± 6, 1200 ± 6, 1350 ± 7, 1500 ± 8, 1650 ± 9 and 1800 ± 9) × 10^−6^ m^3^, provided by a syringe especially used in spirometer calibration procedures. As stated in its manual, the syringe was marked at the points corresponding to volumes of interest, according to Table 1, to perform the calibration. Therefore, it was possible to estimate the systematic error and the range of the random error is expected with 95% of probability, achieving application of the bias correction and collecting information about the uncertainty of the instrument along with its measurement range in future measurements, respectively.

The acceptable limit of error in spirometry for Forced Vital Capacity (FVC) and Forced Expiratory Volume (FEV) is 3.5% of full scale [25]. Therefore, in this work, we considered the maximum error ε equal to 60 × 10^−6^ m^3^, since it represents the limit of 3.4%, satisfying the spirometric conditions.

Considering a small number of repetitions (10 ≤ n ≤ 25), and assuming that the mean of the indications follows an approximately normal distribution, the *t*-Student distribution is used to determine the confidence interval. Due to statistical inference, we have:

(23) ε=tα2s0n.

Ten random measurements were taken to estimate the standard deviation $s_{0}$, which is approximately equal to 98 × 10^−6^ m^3^. For a 95% confidence interval, the significance level α is 0.05, so tα2= 2.2. Thus, a total of 13 measurements (n) should be performed to ensure the statistical significance of the data according to the sampling rules. According to Student’s distribution, we obtained t=2.17, which will be used in subsequent tests [63,64]. The correction is added to the measurements to compensate for the effect of the systematic error. The estimated systematic error corresponds to the average value of the measurement error, i.e., the average of n sensor measurements of the same measurand carried out under repeatability conditions minus the conventional true value of the measurand, provided by a standard or a reference instrument. The correction is equal to the negative of the estimated systematic error [63,64].

The uncertainty of measurement (U) defines an interval about the result of a measurement that may be expected. In this work, it represents the symmetric range of values around the average error where the random error is expected with 95% of probability. As the probability distribution of the sensor measurements follows the Normal distribution (according to the Normal Probability Plot with R-square equal to 0.99927), we considered Student’s distribution to take into account the difference between the standard deviation of the mean and the experimental standard deviation of the mean [63,64]. Thus, the uncertainty of measurement is:

(24) $$U=t_{0.95}\;u,$$

where $t_{0.95}$ is the t-factor from Student’s distribution considering 95% of confidence level; and u is the Type A standard uncertainty, calculated as the experimental standard deviation of the mean.

In this work, the error curve represents the calibration results. It is formed by the center line, which represents the estimated systematic error; and by the upper and lower limits of the range containing the random errors, i.e., the uncertainty of measurement.

### 3.3. Spirometric Tests

The procedures required to perform rescue ventilation in the practice of CPR must follow the parameters of the American Heart Association [65], which establishes a breath every five or six seconds, that is, 10 to 12 breaths during each 60 s. Approximately 500 × 10^−6^ m^3^ of air enters and leaves the lungs of a healthy young adult in a resting state at each respiratory cycle [66]. Therefore, efficient ventilation should provide such a volume of air to the lungs by mouth-to-mouth or using devices for this purpose.

As said before, spirometry is the measure of the air that enters (inspired) and exits (expired) from the lungs. It can be performed during slow breathing or forced expiratory maneuvers. One of the results generated by this technique is an inspired/expired volume versus time graph [25].

**Figure 2:**
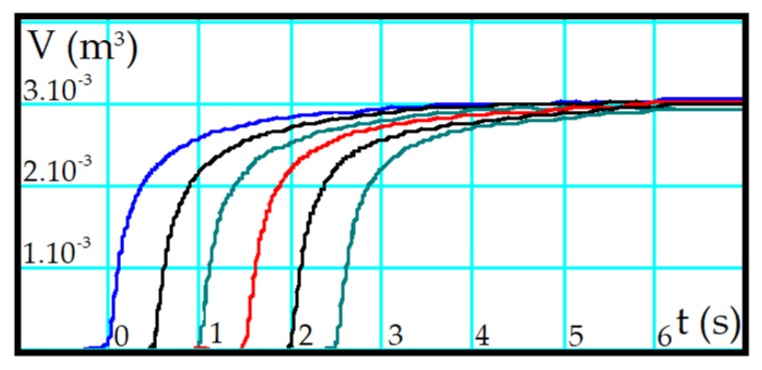
Volume versus Time chart generated by Koko spirometer.

Figure 2 shows, as an example, a result of a real spirometry test performed at the Collective Health Laboratory of the Federal University of Ouro Preto, in a 72-year-old male, 58 kg and 1.68 m; following the stress protocol [25], generated by a commercial instrument (Koko brand). The Koko spirometer utilizes a differential pressure sensor, also known as a pneumotachometers, which measures a small (but measurable) pressure difference around a low-value resistance. As the variations in pressure to be detected are small, the material that constitutes the resistance has a high cost. Furthermore, like other commercial spirometers, it cannot be installed on the CPR training dummy because it takes up a lot of space inside it and the response time does not meet the real-time prerequisites for performing CPR training spirometric feedback. It is also worth noting that the spirometer response automatically correlates the measurement range with the patient’s breathing conditions, what not desirable during CPR, as the goal is to test for optimal ventilatory maneuvers on a cardiorespiratory arrest victim. For volumes between 300 to 600.10^−6^ m^3^, the typical CPR range, the spirometer has difficulty to perform measurements, as this is not the spirometric assessment range, which is usually around 3 to 6 × 10^−3^ m^3^.

Two parameters obtained from these curves are Forced Vital Capacity (FVC) and Forced Expiratory Volume (FEVt). FVC is measured by asking the individual to breathe out until the total lung capacity and expires as rapidly and intensely as possible in a spirometer (Figure 2, FVC = 3 × 10^−3^ m^3^). FEVt can be measured in the FVC maneuver at predefined intervals. In the blue line of Figure 2, FEV is approximately 2.5 × 10^−3^ m^3^ for 1 s, 2.8 × 10^−3^ m^3^ for 2 s, 2.9 × 10^−3^ m^3^ for 3 s, and practically equal to FVC for 4 s. Besides, the FEV value for 1 s should be approximately 80% of the FVC value [25].

From the blue curve of Figure 2, another parameter is obtained: The Medium Forced Expiratory Flow (FEF_25–75%_). To calculate FEF_25–75%_, we mark the points at which 25% and 75% of the FVC were expired on the volume-time curve. A straight line connecting these points is drawn with a duration of 1 s. The vertical distance between the intersection points is FEF_25–75%_ [25].

After calibration, both the YF-S201 sensor and the Koko spirometer were used in a spirometric test, which consisted of applying known air volumes using the syringe: (300 ± 2, 450 ± 3, 600 ± 3, 750 ± 4, 900 ± 5, 1050 ± 6, 1200 ± 6, 1350 ± 7, 1500 ± 8, 1650 ± 9 and 1800 ± 9) × 10^−6^ m^3^. The total volume of air inside the syringe was passed through the spirometer, lifting the curves from the test. Such curves correspond to the volume of the syringe, considering the measurement error.

**Figure 3:**
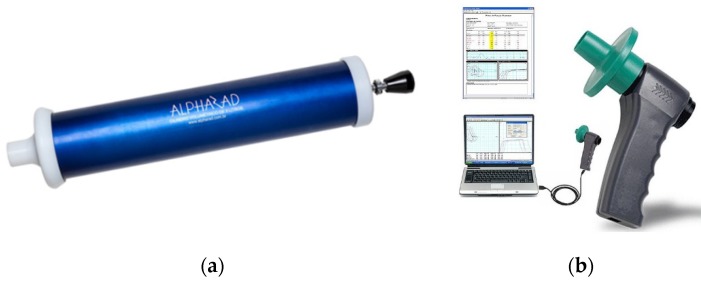
Components for calibrating the spirometer: (**a**) Calibration syringe; (**b**) Koko flow spirometer model 313105.

Beyond the YF-S201 sensor, Figure 3a,b shows the other components used to perform this test: the syringe outlet and the Koko spirometer, respectively. The spirometer shows the uncertainty of 3% or 100 × 10^−6^ m^3^, reproducibility of 0.5% or 150 × 10^−6^ m^3^, volume range of 16 × 10^−3^ m^3^, flow rate 16 × 10^−3^ m^3^/s, and resistance less than 147.1 × 10^3^ Pa/(m^3^s) with the filter.

The measurement results presented by the Koko spirometer were used to verify the quality of the measurements obtained by the system developed in this work, under the same experimental conditions.

### 3.4. Spirometric Feedback in Ventilation Maneuvers during Cardiopulmonary Resuscitation Training

We performed a test using both the sensor developed in this work and the system of the automated dummy manufactured by Laerdal^®^, which uses a linear optical encoder sensor to measure the volume of air entering the lungs. The encoder sensor measures the chest expansion that occurs during the ventilatory maneuver and relates it to the amount of air that has entered in the lung, so it is an indirect measurement. Thus, the range of measured volumes is limited, and it is also impossible to apply spirometric concepts from such indirect measurements.

**Figure 4:**
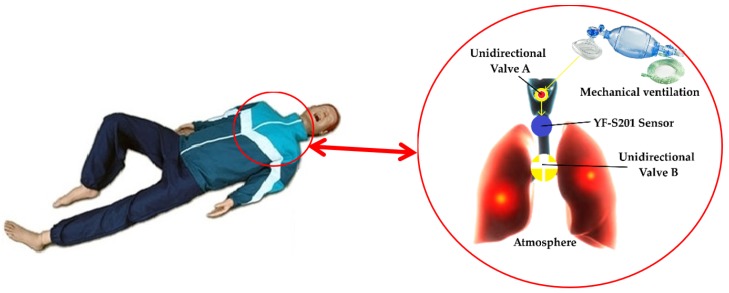
Air route inside the manikin. Adapted from CanStock and SimulaCare.

The sensor installed on the dummy has the configuration of Figure 4, characterizing the system. Air is considered an ideal gas in the temperature range that the sensor works. The one-way valve A is placed in the mouth of the manikin to prevent that contaminated air from returning to the person who is performing the maneuver, due to hygiene. The sensor is coupled between the lung and the one-way valve A, and it has the function of measuring the volume of air entering the lung. The manikin has a single lung with a volume of 3500 × 10^−6^ m^3^ that has the function of storing ventilation air and causing thoracic expansion. The one-way valve B ensures that the amount of air exiting the lung into the atmosphere is less than the amount of air entering the lung, so it is responsible for thoracic expansion.

In 100% of the vital capacity, the inspiratory muscle pressure can reach a maximum of 2942 Pa, and the expiratory muscle pressure can reach at least −2942 Pa [67]. Thus, the maximum pressure difference in the lungs, both expiratory and inspiratory, is 5884 Pa. Most mechanical ventilation devices have a safety valve that operates at a pressure of 4903 to 5884 Pa. Therefore, the maximum pressure in the mechanical ventilation can reach 5884 Pa. Since at the end of the process there is an open tube, the pressure at this point is atmospheric, so the pressure difference at the inlet and outlet of the device does not exceed 6%. In this way, the device installed inside the mannequin addresses the boundary conditions imposed by the theoretical model.

The test procedure involved performing ventilatory maneuvers on the dummy, simulating CPR training, containing inside the arrangement of Figure 4, as well the encoder. The volume of air that enters in the lungs of the dummy and causes chest expansion was measured simultaneously by both the YF-S201 sensor and the encoder.

## 4. Results and Discussion

### 4.1. Calibration and Validation

**Figure 5:**
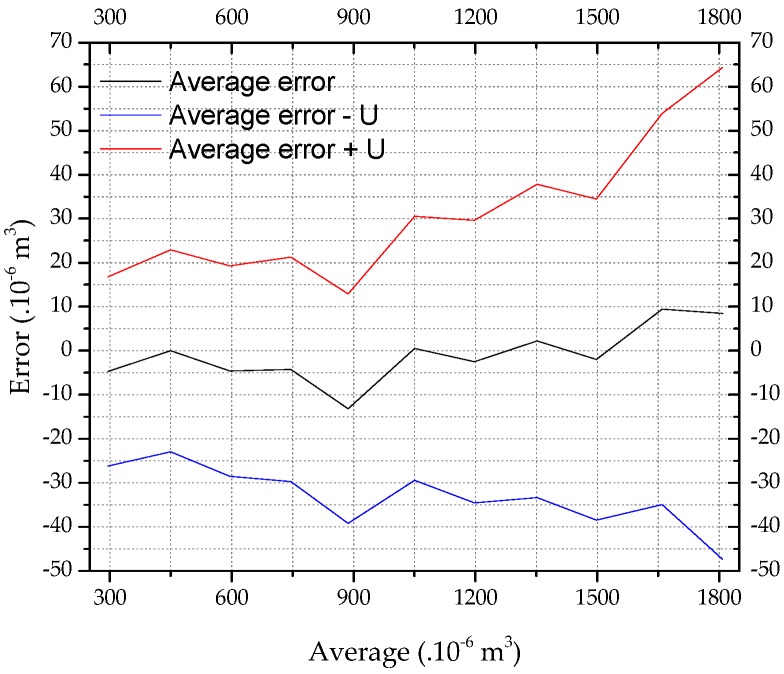
Error curve of the YF-S201 sensor.

Figure 5 shows the error curve for the YF-S201 sensor, considering n=13. There are repeatability and agreement between the results of the measurements performed. Therefore, after applying the bias corrections in the results, the sensor model presents a minimum uncertainty of 22 × 10^−6^ m^3^ for volumes up to 300 × 10^−6^ m^3^, and a maximum uncertainty of 56 × 10^−6^ m^3^ for volumes up to 1800 × 10^−6^ m^3^. Thus, the systematic and random errors were characterized, with a maximum error of 65 × 10^−6^ m^3^ or 3.6%.

After the calibration, the sensor performed the measurements shown in Table 2, using the syringe. The results are according to the spirometric model, and as expected for the performance of the sensor, i.e., the uncertainty is less than 3.4% of the full scale, satisfying the spirometric conditions [25].

### 4.2. Spirometric Tests

**Figure 6:**
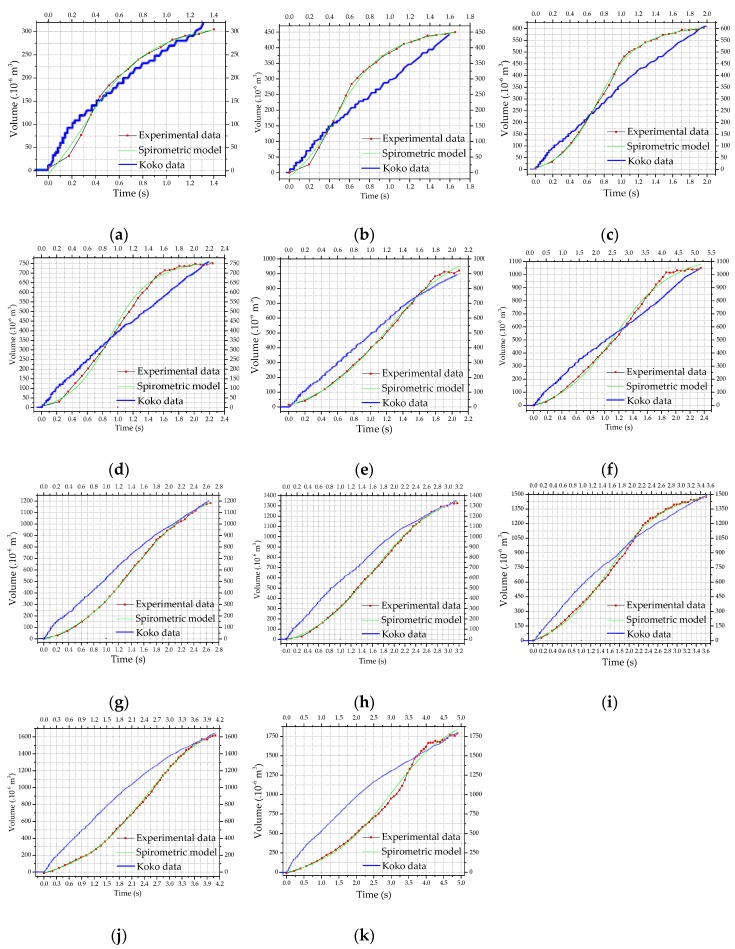
Result of validation of YF-S201 sensor with air: (**a**) 300 × 10^−6^ m^3^; (**b**) 450 × 10^−6^ m^3^; (**c**) 600 × 10^−6^ m^3^; (**d**) 750 × 10^−6^ m^3^; (**e**) 900 × 10^−6^ m^3^; (**f**) 1050 × 10^−6^ m^3^; (**g**) 1200 × 10^−6^ m^3^; (**h**) 1350 × 10^−6^ m^3^; (**i**) 1500 × 10^−6^ m^3^; (**j**) 1650 × 10^−6^ m^3^ and (**k**) 1800 × 10^−6^ m^3^.

Figure 6 shows the measurement results of the YF-S201 sensor (experimental data) and the spirometric model curve obtained from these measurements. We found proximity among the dataset of each graph of Figure 2 and the nonlinear models of Boltzmann’s (BTZ), Logistic (LG), Modified Langevin (ML), Doseresp, Gompertz, Slogistic, and Langmuir EXT 1 (LA). Aiming to fit the dataset to these models, we performed the algorithms of Levenberg Marquardt (LM) and Orthogonal Distance Regression (ODR) for each model. We chose the Langevin model along with the Orthogonal Distance Regression algorithm because it reaches the highest coefficient of determination (R-squared), according to Table 3.

The Langevin function—a simplified version of Brillouin function—is used for classic cases of solid-state physics in quantum treatments. It has applications in paramagnetism [68,69,70,71,72,73] and dielectric properties (permittivity) [74,75,76]. When performing a nonlinear adjustment of experimental data, there may be a need to consider errors in both independent and dependent variables (as in the case of this work). The Orthogonal Distance Regression algorithm [77,78,79] has applications in metrology [80] because it adjusts data with implicit or explicit functions.

Langevin’s function is scale modified to address this application, and the mathematical equation that describes the model is:

(25) $$Y=Y_{0}+C\;[coth\left(\frac{x-x_{c}}{s}\right)-\frac{s}{x-x_{c}}],$$

where $Y_{0}$ is the linear coefficient of equation (m^3^), $x_{c}$ is the central coordinate of the curve (s), C is the amplitude of the curve (m^3^), and s is the scale. To obtain Langevin’s equation, we set the initial guess $Y_{0}\;=\;0$, C = 1, $x_{c}=\;0$ and s = 1.

Table 4 shows the convergence parameters of the non-linear adjustment. It is noteworthy that the R-squared of the calculated model is close to unity, so the modified Langevin mathematical model can be used to describe the spirometric curve and, consequently, the results obtained in this work.

The results of the measurements agree with the conventional values of the measured volume, considering the experimental error. As shown in Table 2, for the reference values of (300 ± 2, 450 ± 3, 600 ± 3, 750 ± 4, 900 ± 5, 1050 ± 6, 1200 ± 6, 1350 ± 7, 1500 ± 8, 1650 ± 9 and 1800 ± 9) × 10^−6^ m^3^, the developed system measured (305 ± 22, 450 ± 23, 603 ± 24, 751 ± 26, 922 ± 27, 1021 ± 30, 1182 ± 33, 1326 ± 36, 1476 ± 37, 1618 ± 45 and 1786 ± 56) × 10^−6^ m^3^ (Figure 6a‒k, respectively).

Comparing the graphs in Figure 6 (Experimental Data and Calculated Spirometric Model) with the spirometric model, from zero to the maximum experimental volume, the behavior follows the spirometer models (Figure 2), characterizing the inspiration. During CPR, there is no muscle activity in the victim’s chest, so the victim’s expiration occurs due to the chest’s weight or due to the resumption of the cardiac massage. Due to these conditions, it is not possible to apply spirometry concepts to the expiration step.

Although the profile of the YF-S201 curves is slightly different from the Koko spirometer results, Table 5 shows that the major part of spirometric results is equivalent. The difference occurs because the Koko spirometer has limitations in use in CPR training, as its measurement range is related to physical breathing parameters such as completely obstructed airway (0‒300 × 10^−6^ m^3^), partially obstructed airway (300‒1000 × 10^−6^ m^3^) or severe disease (200‒2000 × 10^−6^ m^3^). The results of this study refer to cardiorespiratory arrest victims, i.e., a person in conditions of severe disease. However, to perform optimal ventilatory maneuvers during CPR, the result must contain spirometric characteristics such as those obtained by the YF-S201 sensor (Figure 6), whose response does not depend on the measurement range.

From these curves, it was possible to obtain information about FVC, FEV_t=1 s_ and FEF_25–75%_, shown in Table 5. In spirometry, the FEV value in 1 s time is approximately 80% of the FVC value. One ventilation should be done every 6 s in a forced ventilation maneuver. On average, there are 3 s for expiration and 3 s for inspiration. Therefore, for a time of 3 s, FEV always has to be less than FVC, i.e., ventilation should provide FVC within 3 s. It is noteworthy that it happens in the graphs of (300, 450, 600, 750, 900, 1050 and 1200) × 10^−6^ m^3^; around times of 1.40, 1.65, 1.96, 2.25, 2.09, 2.40 and 2.67 s; respectively (Figure 6). In the (1350, 1500, 1650 and 1800) × 10^−6^ m^3^ charts; it occurs around 3.1, 3.61, 4.05 and 4.89 s; respectively (Figure 6). It is also worth noting that the values highlighted in blue in Table 5, measured by the YF-S201 sensor, are different from those obtained by the Koko spirometer. This happens because the higher volumes have a capacity which is not supported by the dynamics of ventilation, because air volumes applied at short intervals cause stomach insufflation to occur, differently from the dynamics of Koko spirometry. If the dynamics of ventilation fail, it is still possible to address the requirements of spirometry applying a faster ventilatory maneuver without stomach insufflation.

It is also worth mentioning that FVC provides the instantaneous maximum expired volume. In mechanical ventilation, it represents the amount of air that was introduced into the lung, and therefore air volume provided in the ventilation. When calculating FEF_25–75%_, note that the values highlighted in green in Table 5 are different from the values measured by the YF-S201 sensor. Meeting the spirometric parameters in this range is difficult, and converges to results from serious diseases, with airway obstruction or dead volumes. To characterize ventilation, performed in humans under the mentioned conditions, the sensor of this work obtains results with adequate spirometric standards [25], unlike the Koko spirometer.

### 4.3. Spirometric Feedback in Ventilation Maneuvers during Cardiopulmonary Resuscitation Training

Comparing measurements performed with the Laerdal^®^ manikin simultaneously with the system of this study, the latter presents a superior performance when compared to the first one, besides a smaller experimental error, according to Table 6. The Laerdal^®^ model is limited to measures below 1000 × 10^−6^ m^3^, and this work is limited to measures below 1800 × 10^−6^ m^3^, therefore, it caters to all devices used in rescue ventilations. Moreover, only when the Laerdal^®^ indicates ≤ 400 × 10^−6^ m^3^, the values are experimentally equal, but below or above this value, there are divergences between the measurements. The Laerdal^®^ model performs indirect measurements of air volume entering the lung based on chest position, which causes errors when the volume is far from 400 × 10^−6^ m^3^. On the other hand, the sensor of this work performs the direct measurement of the air volume, which is much more accurate compared to this kind of indirect measurement.

Comparing the measurements provided by the YF-S201 sensor and the Koko spirometer (Table 5), we observed that the results are experimentally equivalent. Therefore, the YF-S201 achieves the objective of measuring air volume entering the lung of CPR dummies in respiratory maneuvers providing spirometric results. As stated before, the incorporation of sensors such as those presented in [1,2,4,19,27,28,29,30,31,32,33,34,35,36,37,38,39,40,41] is not feasible for this purpose due to, mainly, its high cost.

Another advantage is the simplicity with which measurements are performed, functioning as a noninvasive method that characterizes the ventilation maneuver. The fact that techniques and sensors presented in [1,2,3,4,5,6,7,8,9,10,11,12,13,14,15,16,17,18,19,20,21,22,23,24] require advanced techniques also make their application on dummies unfeasible, due to their complexity and, again, because they have a high cost. Therefore, the alternative presented in this manuscript is attractive for the proposed application because it adds spirometric feedback to ventilation practices in medical simulators using a low-cost sensor that is accord to the application requirements.

The main advantage of the prepared mechanism lies in its cost-effectiveness, the direct measurement of the air entering the lung, and the measurements of spirometric parameters during CPR training. Furthermore, we expect to generate feedback to the users, in future works, as expiration charts based on spirometric models, to bring more realism to the simulations, and innumerable debriefing possibilities.

The spirometric parameters, especially the FVC, along with the graphs generated for debriefing, will allow the student to perform an ideal ventilation maneuver during CPR because the system shows the amount of air that entered the lung and its spirometric input profile from the graphical analysis of the smoothness of the curve. For a more rigid control of the parameters, it is still possible to require time intervals considering the FEVt and to make indirect inference of the airflow using the mean FEV parameter.

## 5. Conclusions

In this work, a sensor was adapted to measure the amount of air supplied to the lungs during ventilation in cardiopulmonary resuscitation (CPR) maneuvers. The calibration and validation of the sensor achieved results that address the CPR requirements. In addition, during the spirometric tests, the system presented the measurement results of (305 ± 22, 450 ± 23, 603 ± 24, 751 ± 26, 922 ± 27, 1021 ± 30, 1182 ± 33, 1326 ± 36, 1476 ± 37, 1618 ± 45 and 1786 ± 56) × 10^−6^ m^3^ for reference values of (300 ± 2, 450 ± 3, 600 ± 3, 750 ± 4, 900 ± 5, 1050 ± 6, 1200 ± 6, 1350 ± 7, 1500 ± 8, 1650 ± 9 and 1800 ± 9) × 10^−6^ m^3^, respectively. Furthermore, we considered both the spirometry and pressure boundary conditions during the experiments using the mannequin lung, according to the results.

The performance of the proposed sensor was compared with a commercial spirometer, and the experimental results were equivalent. The profile of the curves and some measured parameters by the YF-S201 sensor and Koko spirometer are different. The YF-S201 characterizes normal breathing during ventilatory maneuvers while the Koko characterizes breathing from a person with a completely obstructed airway, partially obstructed airway or severe disease during the same maneuvers. After calibration, the YF-S201 sensor showed a minimum uncertainty of 22 × 10^−6^ m^3^ for volumes up to 300 × 10^−6^ m^3^, and a maximum uncertainty of 56 × 10^−6^ m^3^ for volumes greater than 1800 × 10^−6^ m^3^. Thus, the systematic and random errors were characterized, with a maximum error of 65 × 10^−6^ m^3^ or 3.6%.

The experiment confirmed that the measurements can be performed in various simulations using the dummies in conjunction with the sensor. It is a cost-effective alternative, and relatively easy to adapt to different mannequins. The results were based on spirometric models, bringing more realism to the simulations, and bringing numerous possibilities of debriefing. Thus, the sensor has great potential in various future applications.

In future work, we intend to use this sensor on mannequin babies and children. In addition, a supervisory software is being developed for training purposes, and to use in conjunction with the sensor on the manikin. It is also intended to perform the instrumentation of manikins dedicated to the teaching of pulmonary intubation maneuvers and tracheostomy, which the authors believe to be a novelty.

## Figures and Tables

**Table 1:** The relationship between the length of stem and volume provided by the syringe.

**Reference volume** **(×10^−6^ m^3^)** **±0.5%**	0	300	450	600	750	900	1050	1200	1350	1500	1650	1800
**Length of the stem** **(×10^−3^ m)** **±1 × 10^−3^ m**	0	42	64	85	106	127	148	169	191	212	233	254

**Table 2:** Measurements after calibration.

**Reference Volume** **(×10^−6^ m^3^)** **±0.5%**	300	450	600	750	900	1050	1200	1350	1500	1650	1800
**Average Indication** **(×10^−6^ m^3^)**	301	450	601	751	901	1050	1201	1356	1500	1650	1800
**Uncertainty (×10^−6^ m^3^)**	22	23	24	26	27	30	33	36	37	45	56

**Table 3:** R-Square of the non-linear adjustments.

Volume (×10^−6^ m^3^)	R-Square													
	BTZ		LG		ML		Doseresp		Gompertz		Slogistic		LA	
	*L* *M*	*O* *D* *R*	*L* *M*	*O* *D* *R*	*L* *M*	*O* *D* *R*	*L* *M*	*O* *D* *R*	*L* *M*	*O* *D* *R*	*L* *M*	*O* *D* *R*	*L* *M*	*O* *D* *R*
300	I A	N C	I A	N C	I A	0.99999997354852	I A	0.99999996735480	I A	N C	I A	0.99999968697100	I A	N C
450						0.99999998774221		0.99999998313179				0.99999956213635		
600						0.99999999387587		0.99999998738478				0.99999994155838		
750						0.99999997787916		0.99999998623968				0.99999996494737		
900						0.99999999363261		0.99999999373928				0.99999998003168		
1050						0.99999998596560		0.99999998676343				0.99999977894465		
1200						0.99999999843628		0.99999999853441				0.99999994501474		
1350						0.99999999976504		0.99999999797433				0.99999994523958		
1500						0.99999999614389		0.99999999677671				0.99999993443600		
1650						0.99999999808238		0.99999999767503				0.99999680496200		
1800						0.99999998519947		0.99999998367641				0.99999792109656		

*IA—Inadequate accuracy. NC—Not converged.*

**Table 4:** Parameters of convergence of non-linear adjustment applied to the results.

Volume (×10^−6^ m^3^)	Y_0_	x_c_	C	s
300	98 ± 21	0.30 ± 0.07	258 ± 29	0.22 ± 0.02
450	182 ± 10	0.47 ± 0.02	320 ± 14	0.19 ± 0.01
600	284 ± 3	0.72 ± 0.01	372 ± 5	0.18 ± 0.01
750	364 ± 6	0.89 ± 0.02	467 ± 12	0.21 ± 0.02
900	515 ± 12	1.20 ± 0.03	735 ± 39	0.38 ± 0.03
1050	535 ± 9	1.15 ± 0.03	745 ± 33	0.33 ± 0.03
1200	608 ± 4	1.43 ± 0.01	891 ± 15	0.45 ± 0.01
1350	672 ± 5	1.62 ± 0.01	999 ± 19	0.51 ± 0.02
1500	709 ± 6	1.56 ± 0.02	997 ± 14	0.47 ± 0.01
1650	881 ± 6	2.40 ± 0.02	1218 ± 17	0.66 ± 0.02
1800	1006 ± 24	2.98 ± 0.06	1354 ± 54	0.77 ± 0.05

**Table 5:** Comparison between spirometric results of Koko and the sensor developed in this work.

Reference (×10^−6^ m^3^)	Measured Volume (×10^−6^ m^3^)		FVC (×10^−6^ m^3^)		t_FVC_ (s)		FEV_t=1 s_ (×10^−6^ m^3^)		FEF_25–75%_ (×10^−6^ m^3^/s)	
	YF-S201	Koko	YF-S201	Koko	YF-S201	Koko	YF-S201	Koko	YF-S201	Koko
300	305 ± 22	320 ± 23	305 ± 22	320 ± 23	1.4 ± 0.1	1.3 ± 0.1	274 ± 22	260 ± 23	355 ± 26	230 ± 40
450	450 ± 23	450 ± 45	450 ± 23	450 ± 45	1.7 ± 0.1	1.6 ± 0.2	384 ± 23	310 ± 45	500 ± 77	270 ± 81
600	603 ± 24	610 ± 80	603 ± 24	610 ± 80	2.0 ± 0.1	2.0 ± 0.3	463 ± 24	360 ± 80	617 ± 87	330 ± 143
750	751 ± 26	760 ± 100	751 ± 26	760 ± 100	2.1 ± 0.1	2.2 ± 0.3	414 ± 26	390 ± 100	565 ± 78	330 ± 143
900	922 ± 27	890 ± 100	922 ± 27	890 ± 100	2.1 ± 0.1	2.1 ± 0.2	395 ± 27	490 ± 100	585 ± 67	480 ± 150
1050	1051 ± 30	1050 ± 100	1051 ± 30	1050 ± 100	2.4 ± 0.1	2.4 ± 0.2	419 ± 30	490 ± 100	635 ± 78	420 ± 124
1200	1182 ± 33	1200 ± 100	1182 ± 33	1200 ± 100	2.7 ± 0.1	2.7 ± 0.2	334 ± 33	530 ± 100	565 ± 75	490 ± 139
1350	1326 ± 36	1350 ± 100	1326 ± 36	1350 ± 100	3.2 ± 0.1	3.2 ± 0.2	316 ± 36	560 ± 100	597 ± 89	470 ± 129
1500	1476 ± 37	1500 ± 100	1476 ± 37	1500 ± 100	3.6 ± 0.1	3.7 ± 0.2	372 ± 37	570 ± 100	610 ± 96	440 ± 118
1650	1618 ± 45	1650 ± 100	1618 ± 45	1650 ± 100	4.1 ± 0.1	4.2 ± 0.3	197 ± 45	530 ± 100	565 ± 107	430 ± 156
1800	1786 ± 56	1800 ± 100	1786 ± 56	1800 ± 100	4.9 ± 0.1	5.0 ± 0.3	179 ± 56	530 ± 100	525 ± 127	380 ± 136

**Table 6:** Simultaneous measurements of the Laerdal^®^ and YF-S201 sensors.

Laerdal^®^ (×10^−6^ m^3^)	Indicators	This Work (×10^−6^ m^3^)
0	Off	196 ± 2
	Orange	215 ± 2
	Orange	282 ± 2
	Orange	328 ± 3
	Orange	373 ± 3
≤400 ± 60	Orange	419 ± 3
>400 ± 60	Green	557 ± 4
	Green	663 ± 5
≤600 ± 90	Green	851 ± 6
>600 ± 90	Red	1096 ± 2
